# Probe-level estimation improves the detection of differential splicing in Affymetrix exon array studies

**DOI:** 10.1186/gb-2009-10-7-r77

**Published:** 2009-07-16

**Authors:** Essi Laajala, Tero Aittokallio, Riitta Lahesmaa, Laura L Elo

**Affiliations:** 1 Turku Centre for Biotechnology, University of Turku and Åbo Akademi University, Turku, FI-20521, Finland; 2 Department of Mathematics, University of Turku, Turku, FI-20014, Finland

## Abstract

The recent advent of exon microarrays has made it possible to reveal differences in alternative splicing events on a global scale. We introduce a novel statistical procedure that takes full advantage of the probe-level information on Affymetrix exon arrays when detecting differential splicing between sample groups. In comparison to existing ranking methods, the procedure shows superior reproducibility and accuracy in distinguishing true biological findings from background noise in high agreement with experimental validations.

## Abstract 2

A novel statistical procedure is presented that uses probe-level information on Affymetrix exon arrays to detect differential splicing.

## Background

Alternative splicing is the process in which multiple mRNA isoforms are generated from a single gene by selectively joining together exons of a primary transcript in different patterns (see, for example, [1] for a review). Thus, instead of coding a single protein, the same genetic locus may produce a variety of different proteins with different properties and distinct functions in the system. Alternative splicing is emerging as a key mechanism for enabling the vast proteomic diversity of higher organisms from a relatively low number of genes. While genome sequencing projects have revealed that the number of protein-coding genes in an organism does not correlate with its overall cellular complexity (for example, mammalian species have similar numbers of genes to *Arabidopsis thaliana*), alternative splicing has turned out to be more the rule than the exception [2,3]. For instance, genome-wide studies have suggested that up to 92 to 94% of human genes undergo alternative splicing [4]. Tissue-specific gene isoforms are known to play a critical role in the development and proper function of diverse cell types, and disruptions of normal splicing patterns changing the isoform structure have been implicated in various cancer types and other human diseases [5,6]. In particular, a number of genetic point mutations associated with human hereditary diseases have been linked to disrupted splicing [6]. Hence, a comprehensive understanding of disease development requires detailed knowledge of the roles of alternatively spliced genes and their products.

The early genome-wide attempts to detect alternative splicing were mainly based on sequence databases of expressed sequence tags and cDNA [3]. A major drawback of these approaches is that they are highly constrained by the available expressed sequence tag/cDNA sequences, with typically inadequate transcript coverage and only a limited number of cell or tissue sources [3]. Towards the genome-wide identification of functionally relevant alternative splicing events in different cell and tissue types under various conditions, exon microarrays have been introduced [7]. With advanced microarray technology it is now possible to measure all the known and predicted human exons on a single array. For instance, the Affymetrix Human Exon 1.0 ST array contains over 5.4 million probes representing over a million exonic regions (an average of four probes per exon) [8]. In comparison to the conventional gene expression microarrays, which measure transcription at the level of individual genes, the great potential of the exon arrays lies in their ability to provide a finer resolution view of transcription also at the level of individual exons. Hence, the exon arrays enable, for instance, the detection of disease-relevant splicing differences that may be entirely missed in gene-level expression profiling studies [2].

While the detection of differential gene expression between sample groups has been the focus of intensive method development, the detection of differentially spliced transcripts from exon array experiments is still a relatively new area of research. Consequently, the tools for detecting differential splicing are currently much less standardized, including several *ad hoc *methods and algorithms designed for specific analysis tasks or custom platforms only. For example, PAC (pattern-based correlation) identifies splice variants by assuming that, in the absence of splicing, exon expression follows gene expression across the samples [9]. Therefore, it is better suited to studies with multiple different sample types and will generally fail in two-sample cases [9]. Several methods have also been developed for custom microarrays containing splice junction probes. For example, GeneASAP (Generative model for Alternative Splicing Array Platform) attempts to estimate relative expression levels of two isoforms in the same sample with Bayesian learning [10]. For the detection of consistent splicing differences between sample groups, perhaps the most widely used approach currently is the so-called splicing index (SI). The SI approach first normalizes the exon-level expression intensities by the corresponding gene-level summary values and then compares these normalized intensities between the sample groups [11]. The MIDAS (Microarray Detection of Alternative Splicing) algorithm proposed by Affymetrix is based on an analysis of variance (ANOVA) test for differences in the group means of the normalized intensities, being conceptually similar to the SI [9]. Another ANOVA-based method, named ANOSVA (analysis of splice variation), fits a linear model (LM) to the observed data with the aim of identifying non-zero interaction terms between sample groups and exons [12], but this approach did not show favourable performance in the evaluations carried out by Affymetrix [9]. Recently, a procedure called PLATA (Probe-Level Alternative Transcript Analysis) was introduced, which normalizes the expression intensities first probe-wise using the gene-level summary values and then compares the group means of these normalized intensities by considering all the measurements across the probes and samples as independent [13]. Similar probe-wise normalized intensities were recently applied also in [14]. A different type of approach is to formulate the detection of differential splicing as an outlier detection problem, as in REAP (Regression-based Exon Array Protocol) or FIRMA (Finding Isoforms using Robust Multichip Analysis) [15,16]. These approaches aim at identifying exons whose expression deviates significantly from the expected gene-level behaviour. Recent efforts have also been devoted to develop suitable data analysis environments for the exon array studies to handle the massive datasets and their annotations as well as to study the alternative transcripts and their corresponding protein domain architectures [17-19].

In the present work, we introduce a probe-level SI estimation procedure for detecting differential splicing events in Affymetrix exon array studies. With Affymetrix arrays, an important step of the standard SI-based algorithms is the summarization of the probe-level measurements into exon- and gene-level intensities prior to the actual comparisons. However, we and others have shown that the detection of differential gene expression can be markedly improved by considering directly probe-level expression changes instead of such summary intensities [20-24]. Therefore, we hypothesized that a similar strategy would also lead to improvements when detecting differential splicing. The proposed probe-level SI procedure, named PECA-SI, uses a statistical model similar to our previously presented probe-level expression change averaging (PECA) approach, which avoids the need of directly estimating the gene- or exon-level intensities and which does not make any unrealistic assumptions about the independence of the within-individual measurements [20,21]. The benefits of the probe-level detection of differential splicing are demonstrated on both synthetic and real datasets under various circumstances of practical interest with the focus on paired two-group comparisons. In addition to the standard SI calculation procedures using different pre-processing methods (robust multiarray average (RMA), probe logarithmic intensity error model (PLIER)) and statistical algorithms (MIDAS, ordinary or modified *t*-test), the performance of the probe-level SI is compared with two-way ANOVA approaches, closely resembling the ANOSVA procedure, and with the state-of-the-art FIRMA algorithm, which was recently suggested to outperform the SI approach in a simulation study [15].

## Results

We first demonstrate the good performance of the probe-level SI estimation procedure PECA-SI on synthetic data and compare it to standard SI estimation procedures (referred to here as RMA-SI, PLIER-SI, RMA-MIDAS and PLIER-MIDAS), to two-way ANOVA procedures (referred to as RMA-LM and PLIER-LM), as well as to the FIRMA algorithm (see the Materials and methods section for details of the procedures). The benefits of the probe-level approach are then confirmed on multiple publicly available real microarray datasets with different characteristics. The first type of data are from a set of mixture experiments, in which brain and heart samples have been mixed together in different proportions to artificially complicate the detection of the differences between the complex samples [25]. Another dataset contains human brain and tissue pool reference samples that have been hybridized in replicate in two independent laboratories [26]. Finally, we consider measurements from human colon primary tumours and their adjacent normal tissues, being a representative example of a biomedical microarray study with high variability between individuals [27]. In these datasets, we assess the ability of eight different methods, PECA-SI, RMA-SI, PLIER-SI, RMA-MIDAS, PLIER-MIDAS, RMA-LM, PLIER-LM and FIRMA, to reproduce the original detections across various mixture differences, or to detect the same top-ranked candidates between two laboratories or across independent subsamples. The reproducibility reflects the robustness of the methods to identify the relevant splicing events in the presence of confounding factors, laboratory-specific effects or inter-individual variability. The biological relevance of the probe-level procedure is assured by showing its improved ability to detect known brain-specific exons at extremely low false discovery rates, and by demonstrating in the colon cancer data its enhanced ability to discriminate between exons that have been experimentally confirmed with RT-PCR to involve different splice variants and exons that gave negative results in the validations.

### Performance in synthetic data

The simulation study was performed to test the ability of the standard and probe-level SI procedures, the two-way ANOVA approaches, and the recently introduced FIRMA algorithm to detect differential splicing events under controlled settings with known true positives and true negatives. It also allowed us to test the robustness of the methods to multiple exon splicing events within a single gene, which may confound the estimation of the gene-level parameters.

In the synthetic datasets, PECA-SI systematically outperformed the other procedures in detecting the synthetic differential splicing events, as assessed by the receiver operating characteristic (ROC) curves (Table 1). The benefits were largest with the largest numbers of differing exons, supporting the robustness of the PECA procedure in the estimation process. At a typical noise level of *σ *= 0.7 observed in real microarray data [15], the area under the curve (AUC) for PECA-SI remained at 0.92 or above in each case, whereas the AUC values with the other methods decreased from 0.94-0.99 to 0.79-0.88 when the number of differing exons was increased from one to five. The RMA-based methods and FIRMA behaved rather similarly, whereas the relative performance of the PLIER-based methods tended to be poorest when only few exons were differentially spliced or the noise level was increased. As expected, increasing the noise level reduced the performance of all the methods.

**Table 1:** Area under the ROC curve in synthetic data

Differentially spliced exons	Noise level *σ*	PECA-SI	RMA-LM	PLIER-LM	RMA-SI	PLIER-SI	RMA-MIDAS	PLIER-MIDAS	FIRMA
1	0.7	**0.99**	**0.99**	0.98	**0.99**	0.94	**0.99**	0.95	**0.99**
2	0.7	**0.99**	0.98	0.96	0.98	0.94	0.98	0.94	0.98
3	0.7	**0.97**	0.94	0.91	0.93	0.93	0.94	0.93	0.93
4	0.7	**0.94**	0.87	0.83	0.86	0.90	0.90	0.90	0.86
5	0.7	**0.92**	0.83	0.79	0.82	0.88	0.87	0.88	0.81
1	1.0	0.94	0.91	0.89	**0.97**	0.92	0.90	0.85	0.94
2	1.0	**0.94**	0.91	0.88	0.93	0.89	0.91	0.86	0.91
3	1.0	**0.90**	0.86	0.82	0.86	0.82	0.87	0.81	0.84
4	1.0	**0.88**	0.83	0.80	0.84	0.80	0.85	0.79	0.82
5	1.0	**0.74**	0.68	0.68	0.69	0.69	0.72	0.67	0.66

The synthetic data were generated according to Equation 8 at two different noise levels, *σ *= 0.7 or *σ *= 1. The first column indicates the number of synthetic differential splicing events generated within a gene. At each combination of the noise level and the number of differentially spliced exons, 1,000 genes were investigated. In each case, the probe-level PECA-SI procedure (see Equation 6) was compared to the standard SI procedures RMA-SI, PLIER-SI, RMA-MIDAS and PLIER-MIDAS, the two-way ANOVA-based approaches RMA-LM and PLIER-LM, and the FIRMA algorithm. The largest area under the curve (AUC) value across the methods is indicated in bold.

### Reproducibility of detections in the mixture data

**Figure 1:**
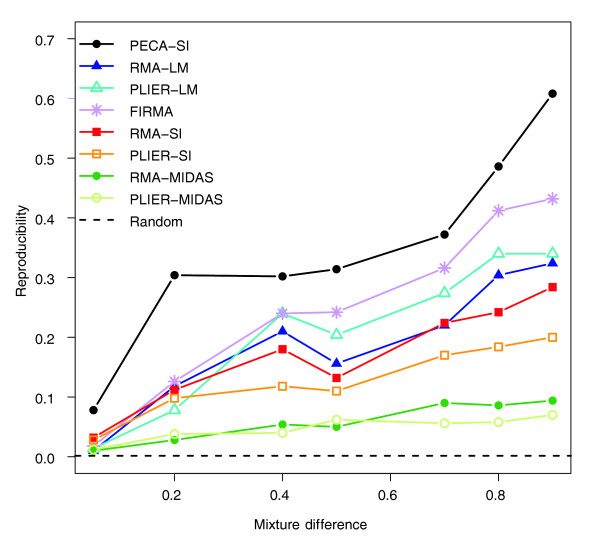
**Reproducibility of detections in the mixture data**. Reproducibility of the probe-level PECA-SI, the standard SI procedures RMA-SI, PLIER-SI, RMA-MIDAS and PLIER-MIDAS, the two-way ANOVA-based approaches RMA-LM and PLIER-LM, and the FIRMA algorithm in detecting differential splicing in the mixture data. The ability of the methods to reproduce the detections from the pure brain and heart samples was studied at various levels of the mixture differences (x-axis). The reproducibility was measured as the overlap of the top-ranked 500 detections between the mixture and pure datasets. At each mixture difference, the same data were analyzed with the different detection methods. Reproducibility in random data is shown as a reference (0.002). Similar results were produced with the top-ranked 100, 1,000, 1,500 and 2,000 detections [see Additional data file 1].

In the mixture data, the different methods were compared in terms of their ability to reproduce the original detections from the pure brain and heart samples using a range of various hybridization mixtures (Figure 1). As expected, with each method the reproducibility decreased when the mixture difference decreased. PECA-SI systematically outperformed all the other methods, producing typically at least twice the number of reproducible detections as the standard SI procedures RMA-SI, PLIER-SI, RMA-MIDAS and PLIER-MIDAS. In addition to FIRMA, the two-way ANOVA-based approaches RMA-LM and PLIER-LM also showed better reproducibility values than the standard SI-based methods, which was somewhat surprising on the basis of the poor ANOSVA result reported in [9]. PECA-SI detected an overlap of approximately 30% between the top-ranked 500 detections already at a mixture difference of 0.2 and this increased to approximately 60% at a mixture difference of 0.9; with FIRMA, RMA-LM and PLIER-LM the percentage remained below approximately 45% even at the largest differences, with RMA-SI and PLIER-SI below 30%, and with the MIDAS approaches below 10%. This suggests that the proposed probe-level procedure can detect the relevant changes much more reproducibly than the conventional approaches even in the presence of confounding factors. The relative performance of the methods remained the same with the top-ranked 1,000, 1,500 and 2,000 detections [see Additional data file 1]. When a lower number of top detections was investigated, the reproducibility values were less stable, which could be attributed to a relatively large number of equally good top candidates in this comparison.

### Reproducibility of detections between laboratories

**Figure 2:**
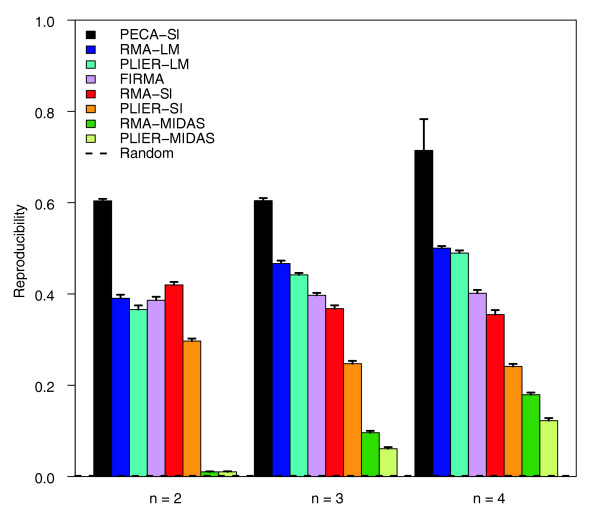
**Reproducibility of detections between laboratories**. Reproducibility of PECA-SI, RMA-SI, PLIER-SI, RMA-MIDAS, PLIER-MIDAS, RM-LM, PLIER-LM and FIRMA in detecting differential splicing between laboratories. The ability of the methods to detect the same candidate splicing events in two independent hybridizations of the same biological samples was investigated at sample sizes of two to four (x-axis). The reproducibility was measured as the overlap of the top-ranked 500 detections between the laboratories. At each sample size, the average reproducibility is shown together with the standard error of the mean (error bars). The same datasets were analyzed with the different detection methods. Reproducibility in random data is shown as a reference (0.002). Similar results were produced with the top-ranked 100, 1,000, 1,500 and 2,000 detections [see Additional data file 2].

The hybridization of the same biological samples in two independent laboratories allowed us to directly assess the reproducibility of the methods across experiments. Since the same biological samples were used in both datasets, the technical laboratory effects could be isolated from the true biological variability. At each sample size, ranging from two to four, PECA-SI systematically showed more reproducible behaviour in each dataset than the other methods (Figure 2). The MIDAS-based approaches performed poorest, especially at the smallest sample sizes, whereas the two-way ANOVA approaches were again at least as good as FIRMA. Also, RMA-SI showed reproducibility values similar to FIRMA and, in this comparison, it outperformed PLIER-SI. In general, the reproducibility of the top candidates increased with increasing sample size. The benefits from larger sample sizes were highest with RMA-MIDAS and PLIER-MIDAS, whereas RMA-SI and PLIER-SI showed even a slight decrease. The relative performance of the methods remained the same with the top-ranked 100, 1,000, 1,500 and 2,000 detections [see Additional data file 2].

### Reproducibility of detections between independent subsamples

**Figure 3:**
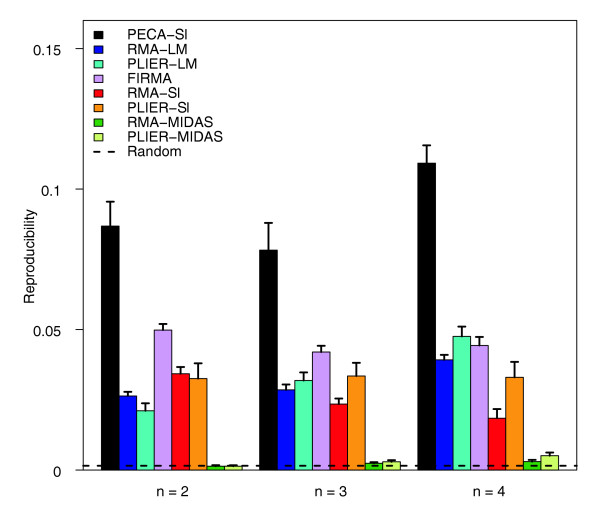
**Reproducibility of detections between independent subsamples**. Reproducibility of PECA-SI, RMA-SI, PLIER-SI, RMA-MIDAS, PLIER-MIDAS, RM-LM, PLIER-LM and FIRMA in detecting differential splicing in the colon cancer data. The ability of the methods to detect the same candidate splicing events in independent subsamples was investigated at sample sizes of two to four (x-axis). The reproducibility was measured as the overlap of the top-ranked 500 detections between the subsamples. At each sample size, the average reproducibility over at least 15 randomly sampled pairs of datasets is shown together with the standard error of the mean (error bar). The same datasets were analyzed with the different detection methods. Reproducibility in random data is shown as a reference (0.002). Similar results were produced with the top-ranked 100, 1,000, 1,500 and 2,000 detections [see Additional data file 3].

In the colon cancer data, the reproducibility of the methods was investigated across independent subsamples of sizes two to four. The aim was to assess the robustness of the methods to detect biologically relevant findings, especially with small sample sizes. Again, PECA-SI was significantly more reproducible than the other methods at each sample size (paired Wilcoxon test, *P *< 0.01), while the MIDAS-based approaches showed the lowest reproducibility values (Figure 3). With these data, FIRMA outperformed RMA-SI; PLIER-SI, RMA-LM and PLIER-LM also showed higher reproducibility values than RMA-SI when the sample size was increased. In general, the reproducibility values were at a similar level to those in the most difficult mixture comparison (mixture difference 0.05), which is in line with the fact that the colon cancer data are from a typical clinical study with large variability between individuals. Increasing the sample size from two to four could not markedly improve the overall level of reproducibility with any of the methods; with the top-ranked 500 detections reproducibility remained, at best, approximately 10% with PECA-SI and was even below 5% with all the other methods. This demonstrates the limitations of the small sample sizes in these types of studies. The relative performance of the methods remained the same with the top-ranked 100, 1,000, 1,500 and 2,000 detections [see Additional data file 3].

### Detection of confirmed splicing events

**Figure 4:**
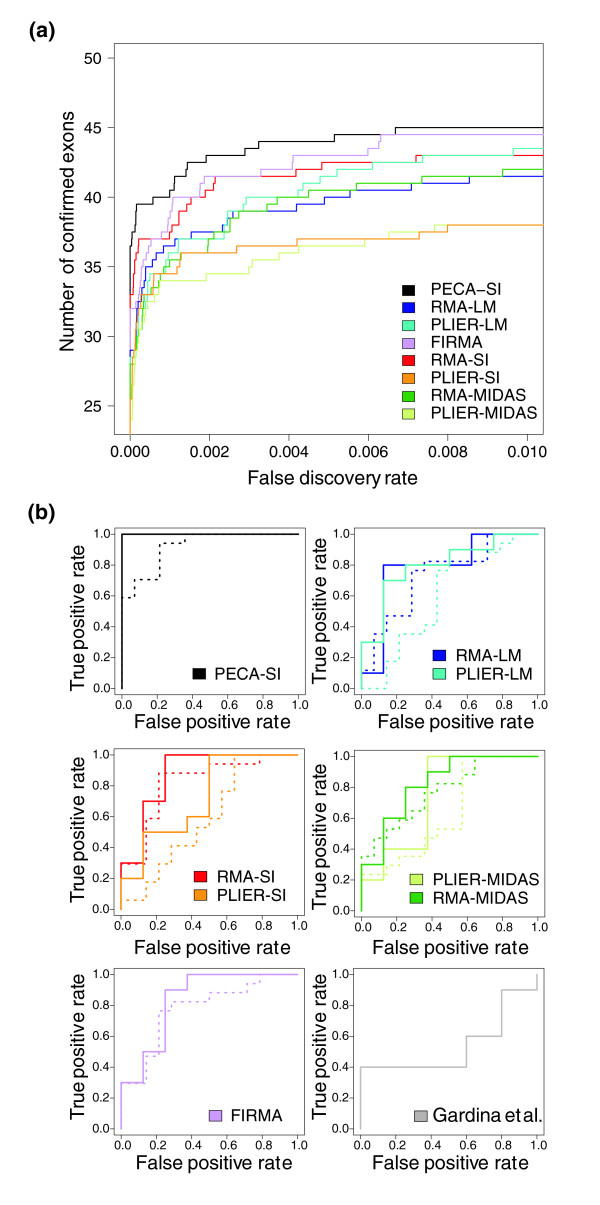
**Detection of confirmed splicing events**. Performance of PECA-SI, RMA-SI, PLIER-SI, RMA-MIDAS, PLIER-MIDAS, RM-LM, PLIER-LM and FIRMA in terms of RT-PCR validations. **(a) **The average number of previously confirmed brain-specific splicing events (y-axis) is shown as a function of the false discovery rate (x-axis) across the two laboratories. **(b) **The ROC curve shows the true positive rate (true positives divided by all positive detections) as a function of the false positive rate (false positives divided by all negative detections) in the colon cancer data. The solid lines correspond to the filtered data (10 positives, 8 negatives), and the dotted lines to the unfiltered data (17 positives, 14 negatives). The results reported in [27] were included as a reference (10 positives, 5 negatives). For the clarity of illustration, the curves for the different analysis approaches are shown in separate graphs. When comparing the curves, the one closest to the upper left corner shows the best performance.

Beyond the reproducibility, we also evaluated the performance of the methods on the basis of RT-PCR-validated differential splicing events to highlight the practical potential of PECA-SI in providing good candidates for further experimental studies. In the between-laboratory comparison data, the different methods were evaluated in terms of a set of exons that were previously confirmed to be differentially spliced between brain and other tissues [11] using a ROC-type approach suggested in [28] together with a randomization procedure. This evaluation supported the biological relevance of the probe-level procedure, as PECA-SI showed the best performance in detecting the known brain-specific exons at a very low false discovery rate (Figure 4a). In addition to RMA-SI and FIRMA, RMA-MIDAS, RMA-LM and PLIER-LM also performed better than PLIER-SI or PLIER-MIDAS in this comparison.

In the colon cancer study, a relatively large set of genes was confirmed with RT-PCR to involve different isoforms in cancer and normal samples. Additionally, several exons gave negative results in the validations, providing a set of true negatives for a ROC analysis. The ROC results further support the benefits of PECA-SI compared to the other methods (Figure 4b). With each method, filtering out genes and exons with low intensities improved discrimination between the confirmed and non-confirmed exons (solid versus dotted lines), although at the same time it reduced the number of validated exon probesets to approximately 60% of the original set (10 confirmed and 8 non-confirmed exon probesets satisfied the filtering criteria). Strikingly, PECA-SI could perfectly separate the confirmed and non-confirmed exons in the filtered data and even in the unfiltered data performed at least as well as the other methods in the ROC analysis after filtering. Comparison of the methods with the original list of the top-ranked 200 detections reported in [27] suggested that the stringent filtering criteria applied in the original study could not improve the discrimination between the true positives and true negatives. Instead, their approach gave the poorest ROC results in this comparison.

The ranks of the confirmed probesets ranged widely in the genome-wide comparison, as observed also in [15]. In general, PECA-SI tended to give relatively high ranks. For instance, in the cancer data after filtering, two validated exons were already found among the top ten detections with PECA-SI (ACTN1 probeset 3569830 rank 1, COL6A3 probeset 2605390 rank 7), whereas the best-ranking validated exon was ranked 28th with RMA-SI (ACTN1 probeset 3569830), 35th with PLIER-SI (CALD1 probeset 3025632), 50th with RMA-LM (MAST2 probeset 2334499), 82nd with PLIER-LM (MAST2 probeset 2334499), and 26th with FIRMA (ACTN1 probeset 3569830). With the MIDAS-based approaches, which were also applied in the original study [27], the best-ranking confirmed exon was ranked 5th with RMA-MIDAS (ACTN1 probeset 3569830) and 4th with PLIER-MIDAS (COL6A3 probeset 2605390).

## Discussion

In the present work, we have demonstrated the clear benefits of using directly all the available probe-level data when detecting consistent differential splicing events between sample groups. The benefits of PECA-SI accumulate from two sources: an improved estimate of the gene-level signal log-ratio; and an improved estimate of the exon-level statistic determined on the basis of its probe-level values [see Additional data file 4]. In contrast to the conventionally utilized summary intensities, which yield a single gene/exon-level value of a statistic, the probe-level approach takes advantage of its probe-level distribution, improving thereby the reliability of the estimation process. Moreover, the proposed PECA procedure simplifies the estimation by avoiding the determination of the probe affinities (Equation 3 versus Equation 1 and Equation 5 versus Equation 4 in the Materials and methods section). The superior performance of the probe-level PECA-SI over the variety of previously proposed methods is shown here systematically on both synthetic and real datasets in various practical comparisons. Importantly, PECA-SI was able to detect confirmed differentially spliced exons in the complex colon cancer study, demonstrating its high potential also in real biomedical applications.

We focused here mainly on the rankings of the exons, since in practice the ranking determines which genes/exons will be considered for further experiments. While the statistical significance of the detections can be calculated similarly as in the case of detecting differential gene expression, reasonable multiple testing correction is even more challenging due to a huge number of exons tested in parallel on the arrays and the fact that they are highly non-independent [19]. In particular, nonparametric permutation approaches become computationally very intensive.

A critical final step in an exon array study is the evaluation of the relevance of the detected exons. While PECA-SI can improve the reliability of the detections, there remain cases in which it is difficult to distinguish between true splicing events and differences caused by poorly designed probesets. In particular, although essential for the discovery of novel splicing events, the large number of speculative probesets on the array necessitates careful attention [11]. For instance, many predicted probesets may interrogate regions that are not actually transcribed at all and will, therefore, be falsely detected as differentially spliced. Another type of false detection arises from probesets showing increased expression due to cross-hybridization with another gene. To guard against such false detections, the exon lists can be filtered using various criteria, such as low intensity or probe specificity, and, finally, by visually inspecting the intensities of the best candidates within the genomic context. Ultimately, the detections can be confirmed using an independent experimental technology, such as RT-PCR. As the experimental validations are laborious, the filtering criteria should be a balance between the available resources and the aim of the study to extend the limits of the detections. Future improvements in the accuracy and coverage of annotations are likely to improve also the reliability of the exon array results. The proposed probe-level procedure is applicable to any existing or future annotation scheme.

In addition to the annotation accuracy, another challenging issue in the detection of differential splicing is the complexity of the splicing process. Several types of splicing events have been observed, such as exon inclusion/exclusion, alteration of exon length, intron retention or alternative promoter or polyA sites [1]. The different transcript variants are produced combinatorially through these events and multiple different isoforms of the same gene may occur in a single sample. A limitation of the SI approaches as well as the FIRMA model is that they cannot truly capture complex transcript patterns involving multiple isoforms. Instead, they may, in the worst case, produce erroneous results if the multiple isoforms share overlapping regions [29]. Hence, development of more complex measures of differential splicing may be required as the understanding of the splicing process evolves. As the aim of the present study was to demonstrate the benefits of using directly the probe-level data in detecting differential splicing, SI was chosen as a widely used and straightforward approach. In general, the proposed probe-level procedure is not limited to SI calculations only but could be extended to other types of probe-level statistics as well.

A comprehensive characterization of the transcriptome with the different splice variants and the assessment of their functional roles, using, for example, large-scale small interfering RNA screens, can open up new perspectives on how different cellular processes are regulated in normal and disease states [2]. In particular, alternative splicing signatures hold a great promise to provide novel diagnostic and prognostic tools for many diseases [6]. This was supported, for instance, by the recent study of prostate cancer, demonstrating that the detection of splice variants can indeed permit more reliable discrimination between normal and tumour tissues than the detection of gene-level differences in the same samples [30]. Moreover, the ability to measure individual exons and isoforms provides new possibilities for combining the transcriptomic and proteomic measurements, which have typically shown little correlation in the conventional gene-level analyses [31]. Providing an additional layer to the gene regulation network, exon-level analysis of expression is likely to be an intensive focus of research in the near future. An important future goal is the effective integration of the exon-level data with all the available data from other levels of the systems, such as protein abundance measurements or protein-protein, domain-domain or protein-DNA interactions.

## Conclusions

Alternative splicing has appeared as a key mechanism by which higher organisms increase their proteomic and functional diversity. Therefore, characterization of the full repertoire of relevant transcript variants and their specific roles in the cells is essential for a detailed understanding of various normal and disease states. With the massive datasets produced by exon microarrays consisting of millions of data points per sample, effective methods are needed to dissect the true biological findings from background noise. In the present work, we introduced a novel probe-level procedure for ranking exons on the basis of differential splicing in Affymetrix exon array studies. In comparison to existing ranking methods, the proposed PECA-SI procedure showed superior performance systematically under various practical comparisons on synthetic and real datasets. In particular, significant improvements were achieved in the reproducibility of the detections even in the presence of confounding factors. The biological relevance of the procedure was finally confirmed by its enhanced ability to discriminate between true positive and true negative detections as assessed experimentally by RT-PCR.

## Materials and methods

### Detection of differential splicing

#### Intensity model

The widely used model for the normalized logarithmic intensity of a probe *k *corresponding to a probeset *g *(conventionally a gene) in a sample *u *is defined as:

(1)  (Equation 1)

where the parameter *μ*_ug _denotes the expression level of the probeset *g *in the sample *u*, *θ*_gk _accounts for the fact that different probe sequences can have different binding properties, and *ε*_ugk _is the measurement error [32]. Since microarray data typically contain several outliers due to, for instance, bad-quality probes, false annotations or alternative splicing, robust estimation methods are often applied [32,33].

#### Splicing index

The standard SI procedure considers Equation 1 at two levels: the genes and the exons [11]. The underlying assumption is that the number of differentially spliced exons is much smaller than the total number of exons in the gene. To calculate the SI value for an exon *e *corresponding to a gene *g *in a sample *u*, the probe-level expression intensities are first summarized into an exon-level intensity  and the corresponding gene-level intensity . The exon intensity is then normalized by the gene intensity, producing the normalized intensity (NI) log_2 _. Finally, the SI between two samples *u *and *v *is defined as the log-ratio between their NI values:

(2)  (Equation 2)

In case of replicated samples in the two sample groups under comparison, the ordinary or a modified *t-*test can be applied to the NI or SI values to identify exons that show statistically significant differences between these groups [11,27].

#### Probe-level expression change averaging

It can be observed that the probe effect *θ*_gk _in Equation 1 is cancelled out if relative expression levels between two samples *u *and *v *are considered instead of their absolute signal intensities. This simplifies the model to:

(3)  (Equation 3)

which allows the probeset-level expression change *μ*_(uv)g _= *μ*_ug _- *μ*_vg _to be estimated directly using, for instance, the median over the probes. This type of probe-level expression change averaging approach PECA has been shown to improve the detection of differential expression in gene expression microarray studies [20]. Moreover, in case of replicated samples, it has been shown that it is beneficial to also apply a similar probe-level procedure to other measures of differential expression, such as a *t-*type statistic between sample groups [20,23].

#### PECA splicing index

To apply a PECA-type procedure to the SI calculations, a probe-level SI needs to be defined. Therefore, we consider a modified version of Equation 1 that takes into account the potential differences in the exon inclusion rates:

(4)  (Equation 4)

Here, *α*_*uge *_denotes the effect of an exon *e *corresponding to a gene *g *in a sample *u*. In light of this model, the logarithmic *NI*_*uge *_value can be viewed as an estimate of the exon effect *α*_*uge*_. Comparing the expression levels between two samples *u *and *v *gives:

(5)  (Equation 5)

where *μ*_(uv)g _= *μ*_ug _- *μ*_vg _is the gene-level expression change, while *α*_(uv)ge _= *α*_uge _- *α*_vge _corresponds to the *SI*_(*uv*)*ge *_in Equation 2. Hence, a natural definition of the probe-level SI of a probe *k *is:

(6)  (Equation 6)

where the gene-level expression change  is estimated from Equation 3. Since our ultimate goal is to detect systematic splicing differences across biological (paired) replicates, the ordinary or modified *t-*statistic is calculated separately for each probe. The exon-level statistic is finally determined as the median over the probe-level values of the statistic.

#### FIRMA algorithm

The recently introduced FIRMA approach was considered in the present work as a state-of-the-art reference method, although it was originally designed for situations without replication [15]. In the FIRMA algorithm, the parameters *μ*_ug _and *θ*_gk _are estimated from Equation 1 using iteratively weighted least squares estimation [15]. The detection of alternative splicing is then formulated as an outlier detection problem, where the residual  is evaluated for each probe *k*. The final FIRMA score of an exon is defined as the median residual over the probes within the particular exon probeset divided by their median absolute deviation. For comparability, the FIRMA scores were also subjected to a *t-*type statistic to identify consistent splicing differences across replicates.

#### Two-way ANOVA approaches

A two-way ANOVA can be used to model the observed logarithmic intensities of a given gene as a combination of two factors, exon and sample group:

(7)  (Equation 7)

Here, x_uec _denotes the intensity of an exon *e *of a sample *u *in a sample group *c*. The term *μ *represents the baseline intensity of the particular gene, the terms *α*_*e *_and *β*_c _represent the linear contributions of the exon *e *and the sample group *c*, respectively, and the term *γ*_*ec *_represents their interaction; *ε*_uec _is the error term. Differential alternative splicing between sample groups can be detected by assessing the significance of each interaction term *γ*_*ec *_[12,26]. Here, the significance was assessed similarly as in [12] using a *t-*test, where the numerator and the denominator of the test statistic are the estimated coefficient and its standard error, respectively, and there are n - *ν *- 1 degrees of freedom, where *n *is the sample size and *ν *is the number of terms in the statistical model.

#### Filtering

When detecting differential splicing events, special care should be taken of genes and exons that are not expressed. To avoid spurious detections, a gene is often required to be expressed in both sample groups and an exon in at least one of the sample groups [8]. If the gene is expressed in only one group, then there is no true differential splicing between the groups, although SI may detect alternative splicing events in the expressed group. On the other hand, if the exon is not expressed in either of the groups, then SI will detect the gene-level differences between the groups instead of differential splicing. To consider the effect of non-expressed genes and exons, we also evaluated the methods after applying a filtering procedure. Following the approaches of [15,27], we defined a probe as present in a sample group if its expression level in at least half of the samples in that group was larger than a predefined threshold. The threshold was determined as the overall probe median as in [34]. A gene was selected for further analysis only if at least half of its probes were present in both sample groups, resembling the procedure of [27]. Similarly, an exon was selected only if it contained at least three present probes in either of the sample groups.

#### Implementation

The PECA-SI, RMA-SI and FIRMA calculations were performed in R using the package aroma.affymetrix, which is specifically designed to handle large datasets produced in high-throughput experiments [17]. For PECA-SI, the data were pre-processed using the quantile normalization method as in the previous PECA applications [20,21]. The gene- and exon-level changes were calculated as the medians over the probes. For RMA-SI, the gene- and exon-level intensities were estimated using the RMA procedure. The FIRMA model was fitted using the default implementation in the aroma.affymetrix package together with logarithmic transformation. To detect consistent splicing differences between sample groups, ordinary or modified *t-*statistics were determined. With small sample sizes (*n *< 10), the modified *t-*statistic in the Bioconductor limma package was utilized [35,36]. With larger sample sizes (*n *≥ 10), the ordinary *t*-statistic was calculated. In the present work, we focused on paired two-sample designs and, hence, the *t-*statistics were calculated using the SI values from the paired samples.

The RMA-MIDAS and PLIER-MIDAS analyses were performed using the Affymetrix Power Tools software provided by the array manufacturer [37]. In addition to the RMA procedure, the pre-processing of the data was also done using the PLIER. The standard PLIER algorithm was used to estimate the exon-level intensities, whereas its iterative version (IterPLIER) was applied to derive the gene-level intensities, similarly as in [27]. Default parameters were used in each of the algorithms. For the PLIER-SI and the two-way ANOVA analyses, referred to as RMA-LM and PLIER-LM, the RMA or PLIER pre-processed data from the Affymetrix Power Tools software were imported into R. For PLIER-SI, the SI-calculations were performed in R similarly as with the RMA-SI procedure. For RMA-LM and PLIER-LM, the exon-sample group interactions were assessed using the function lm in R.

Gene-level probeset definitions based on the human Ensembl build 49 were downloaded from the aroma.affymetrix website [38]. Within these probesets, the original exon-level probeset definitions of Affymetrix were retained.

The R package PECA implementing the PECA-SI procedure is available from our website [39].

### Datasets and evaluation criteria

#### Synthetic data

Synthetic data were generated using a similar model as in [15], featuring additive background, multiplicative noise and probe-specific affinities. More specifically, the intensity of a probe *k *for a gene *g *in a sample *u *was simulated from the model:

(8)  (Equation 8)

where log_2 _(*B*_*gk*_) ~ *N*(5,0.35^2^) is the background, *μ*_*ug *_~ *N*(7,1.5^2^) is the expression level in sample *u*, *θ*_*gk *_~ *N*(0,3^2^) is the probe affinity, *ε*_*ugk *_~ *N*(0, *σ*^2^) is the measurement error at noise level *σ *= 0.7 or *σ *= 1, and *I*_*ugk *_is an indicator function determining whether the exon is included in the transcript (*I*_*ugk *_= 1) or not (*I*_*ugk *_= 0). The parameters were taken from [15]. Additionally, a higher noise level *σ *= 1 was considered. The exon structure of the genes was taken from the Affymetrix Human Exon 1.0 ST array based on the Ensembl annotations. For each gene, one to five differentially spliced exons between two groups of ten samples were generated. In total, 10,000 genes were considered, 1,000 at each parameter setting (number of differentially spliced exons and the noise level). Since the true differential splicing events in the synthetic data are known, the performance of the methods was assessed in terms of their ROC curves. A ROC curve determines the true positive rate of a method as a function of the false positive rate when the number of top-ranking exons is varied. To summarize each ROC curve into a single value, the AUC was calculated.

#### Mixture data

The mixture data were downloaded from the Affymetrix website [25]. In these data, total RNA from brain and heart were mixed together in nine different proportions (sample sets mix1, mix2, ..., mix9 in [25]) and hybridized in triplicate on the Affymetrix Human Exon 1.0 ST arrays. Even if the true expression changes are not known, it is known that the detections made when comparing the pure brain and heart samples (mix1 versus mix9) should also be identified in the mixtures. Thus, the performance of the methods can be evaluated in terms of their capability to reproduce the original pure sample detections across a range of mixture differences. In the present work, seven comparisons with different levels of mixture differences were investigated: 0.05 (mix2 versus mix3), 0.2 (mix2 versus mix4), 0.4 (mix3 versus mix5a), 0.5 (mix4 versus mix6), 0.7 (mix2 versus mix6), 0.8 (mix3 versus mix7), 0.9 (mix2 versus mix8). For details of the particular mixtures, the reader is referred to [25]. The reproducibility was defined as the overlap of the top-ranked *k *detections between the mixture and the pure data, *k *= 100, 500, 1,000, 1,500, 2,000.

#### Between-laboratory comparison data

The between-laboratory comparison data [GEO:GSE13072] contain measurements from human brain and tissue pool reference samples [26]. Five technical replicates of both sample types were hybridized independently in two different laboratories, resulting in a total of ten arrays per laboratory. In addition to the whole datasets, we investigated the performance of the different splicing detection methods in all the possible subsamples of sizes two to four. In each case, two replicate estimates of differential splicing across the exons were obtained corresponding to the same samples in the different laboratories. Similarly as in the mixture data, the agreement of the top-ranked *k *candidates was examined at *k *= 100, 500, 1,000, 1,500, 2,000. To assure the biological relevance of the detections, the performance of the different methods was further evaluated using a set of brain-specific exons previously confirmed with RT-PCR [11]. Of these confirmed exons, 51 matched the probesets in our analysis. The evaluation was performed in a ROC-type manner by assessing the number of true positives as a function of the false discovery rate, similarly as suggested in [28]. Since there was no sufficient set of true negatives available, false discovery rates were estimated using a randomization procedure. More specifically, random datasets were generated by repeatedly permuting the sample labels 100 times; each of the procedures was then applied to these randomized datasets; and the false discovery rate of a procedure was finally estimated as the expected proportion of falsely called exons among all the positive predictions at a particular cutoff level of the statistic.

#### Colon cancer data

The colon cancer data of [27] involve ten matched pairs of human colon primary tumour and adjacent normal tissue (20 total RNA samples), being a representative example of a real biomedical microarray study. As opposed to the other datasets, the colon cancer data are expected to be noisy due to, for instance, different stages of the cancer progression (poorly/moderately/well differentiated tumours), heterogeneous tissue samples, and high variability between the individuals [27]. To evaluate the methods in these data, we first assessed the reproducibility of the top-ranked candidates detected in independent subsets of two to four randomly selected sample pairs. This gives indications of the robustness of the procedure, as biologically relevant splicing events should be detected across replicates. Similarly as before, the reproducibility was determined as the overlap of the top-ranked *k *candidates, *k *= 100, 500, 1,000, 1,500, 2,000. At least 15 pairs of independent subsets were considered at each sample size. In order to compare how accurately the methods could discriminate the true splicing differences from background noise, we utilized the relatively large set of RT-PCR validations performed on the same data [27]. Of the exons included in the RT-PCR runs and matching the probesets in our analyses, 17 were confirmed to have cancer-specific splicing and 14 showed clearly negative results by RT-PCR. To assess the performance of the methods with respect to the validated exons, a ROC analysis among them was conducted.

## Abbreviations

ANOSVA: analysis of splice variation; ANOVA: analysis of variance; AUC: area under the curve; FIRMA: Finding Isoforms using Robust Multichip Analysis; LM: Linear Model; MIDAS: Microarray Detection of Alternative Splicing; NI: normalized intensity; PECA: probe-level expression change averaging; PLIER: probe logarithmic intensity error model; RMA: robust multiarray average; ROC: receiver operating characteristic; SI: splicing index.

## Authors' contributions

EL performed the testing. TA participated in evaluating the results and drafting the manuscript. RL provided biological motivation and insight into the project. LLE initiated and supervised the study, developed the probe-level algorithm and its R implementation, participated in the testing and drafted the manuscript. All of the authors read and approved the final manuscript.

## Additional files

The following additional data are available with the online version of this paper: figures showing reproducibility of detections in the mixture data (Additional file 1); figures showing reproducibility of detections between laboratories (Additional file 2); figures showing reproducibility of detections between independent subsamples (Additional file 3); figures showing accumulated benefits of probe-level estimation in the mixture data (Additional file 4).

## Supplementary Material

Additional data file 1 Reproducibility of PECA-SI, RMA-SI, PLIER-SI, RMA-MIDAS, PLIER-MIDAS, RM-LM, PLIER-LM and FIRMA in detecting differential splicing in the mixture data. The ability of the methods to reproduce the detections from the pure brain and heart samples was studied at various levels of the mixture differences (x-axis). The reproducibility was measured as the overlap of the top-ranked *k *detections between the mixture and pure datasets; *k *= 100, 1,000, 1,500, 2,000. At each mixture difference, the same data were analyzed with the different detection methods. Reproducibility in random data is shown as a reference (0.0003, 0.003, 0.005 and 0.006 with the top 100, 1,000, 1,500 and 2,000 detections, respectively). Click here for file

Additional data file 2 Reproducibility of PECA-SI, RMA-SI, PLIER-SI, RMA-MIDAS, PLIER-MIDAS, RM-LM, PLIER-LM and FIRMA in detecting differential splicing between laboratories. The ability of the methods to detect the same candidate splicing events in two independent hybridizations of the same biological samples was investigated at sample sizes two to four (x-axis). The reproducibility was measured as the overlap of the top-ranked *k *detections between the laboratories; *k *= 100, 1,000, 1,500, 2,000. At each sample size, the average reproducibility is shown together with the standard error of the mean (error bars). The same datasets were analyzed with the different detection methods. Reproducibility in random data is shown as a reference (0.0003, 0.003, 0.005 and 0.006 with the top 100, 1,000, 1,500 and 2,000 detections, respectively). Click here for file

Additional data file 3 Reproducibility of PECA-SI, RMA-SI, PLIER-SI, RMA-MIDAS, PLIER-MIDAS, RM-LM, PLIER-LM and FIRMA in detecting differential splicing in the colon cancer data. The ability of the methods to detect the same candidate splicing events in independent subsamples was investigated at sample sizes two to four (x-axis). The reproducibility was measured as the overlap of the top-ranked *k *detections between the subsamples; *k *= 100, 1,000, 1,500, 2,000. At each sample size, the average reproducibility over at least 15 randomly sampled pairs of datasets is shown together with the standard error of the mean (error bars). The same datasets were analyzed with the different detection methods. Reproducibility in random data is shown as a reference (0.0003, 0.003, 0.005 and 0.006 with the top 100, 1,000, 1,500 and 2,000 detections, respectively). Click here for file

Additional data file 4 Illustration of the benefits gained by the probe-level estimation of PECA-SI in the mixture data. **(a) **Probe-level PECA improves the estimation of the gene-level signal log-ratios as compared to the standard procedure of using the RMA-normalized summary intensities. Each mixture contains three technical replicates, which are assumed to produce the same results across the arrays. To asses the performance of the PECA- and RMA-based estimation, we repeatedly calculated signal log-ratios between pairs of samples from distinct mixtures and then assessed the reproducibility of the estimates between the technical replicates using the Spearman correlation. The average reproducibility at various mixture differences (x-axis) is shown together with the standard error of the mean (error bars). It can be observed that the probe-level PECA produced systematically higher reproducibility values than RMA (paired Wilcoxon test, *P *< 10^-6^).**(b) **The probe-level PECA-SI improves the estimation of the exon-level statistic for differential splicing as compared to the standard summary intensity-based procedures (RMA-SI and RMA-MIDAS) or to a probe-level procedure, in which PECA is first applied separately to calculate the gene- and exon-level changes and these summarized changes are then used to calculate the statistic for SI (sPECA-SI). Similarly as in Figure 1, the ability of the methods to reproduce the detections from the pure brain and heart samples was studied at various levels of the mixture differences (x-axis). The reproducibility was measured as the overlap of the top-ranked 500 detections between the mixture and pure datasets. At each mixture difference, the same data were analyzed with the different detection methods. Reproducibility in random data is shown as a reference (0.002). Click here for file
