# Supplementary figures and images for: Probe-level estimation improves the detection of differential splicing in Affymetrix exon array studies
Source: Genome Biol. 2009 Jul 16;10(7):R77. doi: 10.1186/gb-2009-10-7-r77 (PMC2728531; doi:10.1186/gb-2009-10-7-r77)

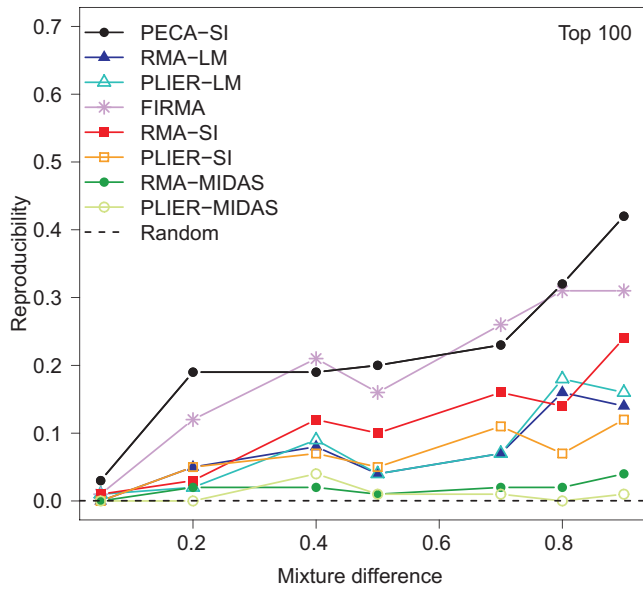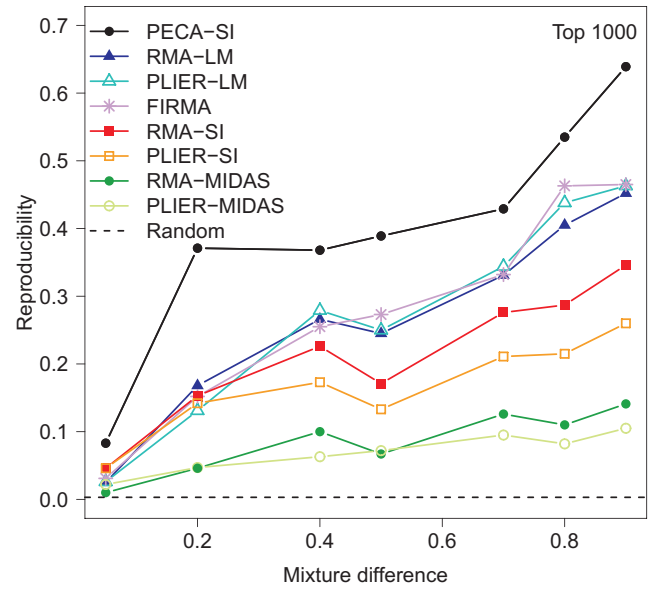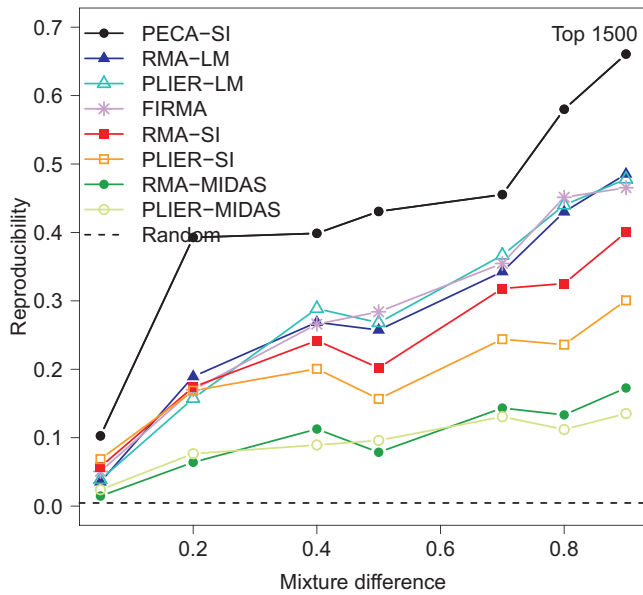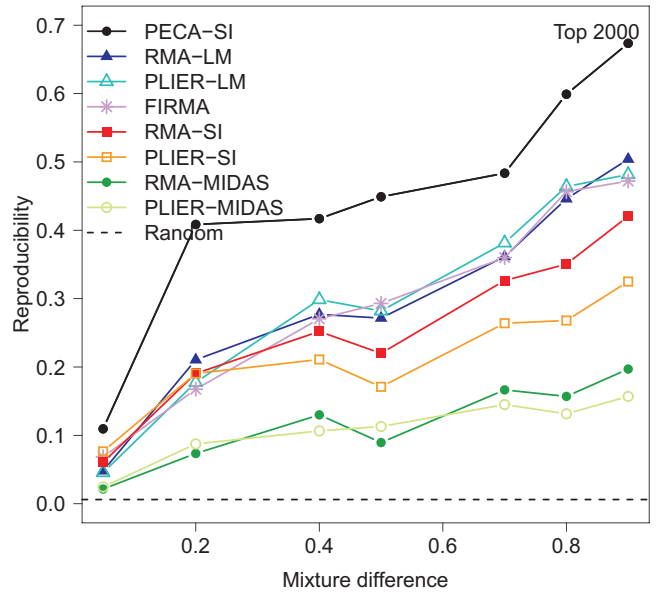

Supplement: Additional data file 1 — Reproducibility of PECA-SI, RMA-SI, PLIER-SI, RMA-MIDAS, PLIER-MIDAS, RM-LM, PLIER-LM and FIRMA in detecting differential splicing in the mixture data. The ability of the methods to reproduce the detections from the pure brain and heart samples was studied at various levels of the mixture differences (x-axis). The reproducibility was measured as the overlap of the top-ranked k detections between the mixture and pure datasets; k = 100, 1,000, 1,500, 2,000. At each mixture difference, the same data were analyzed with the different detection methods. Reproducibility in random data is shown as a reference (0.0003, 0.003, 0.005 and 0.006 with the top 100, 1,000, 1,500 and 2,000 detections, respectively). [file gb-2009-10-7-r77-S1.pdf]

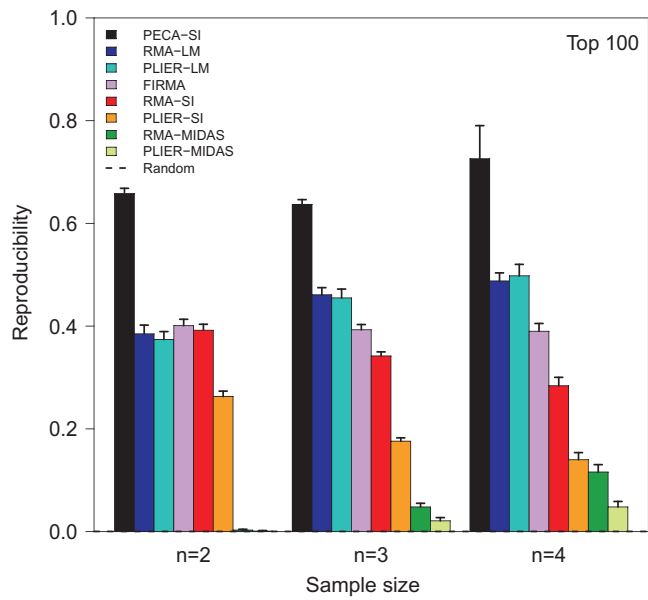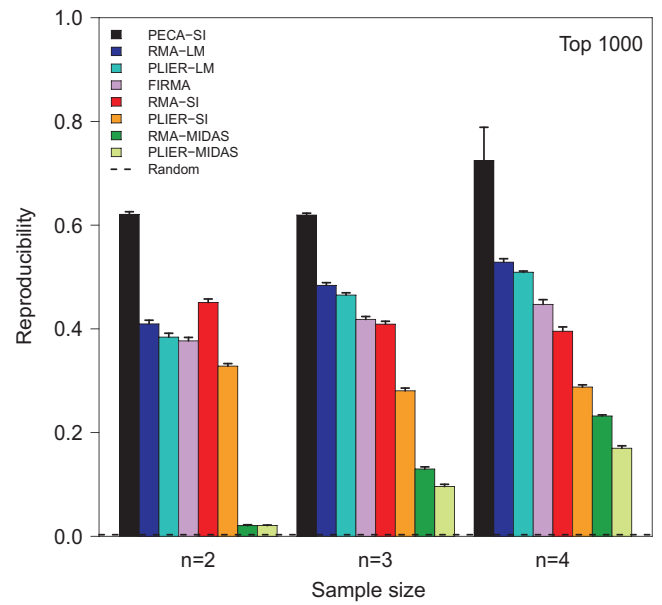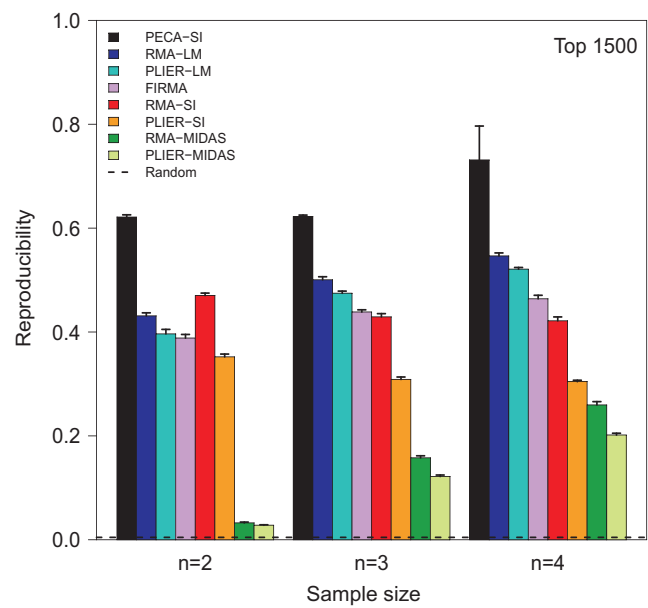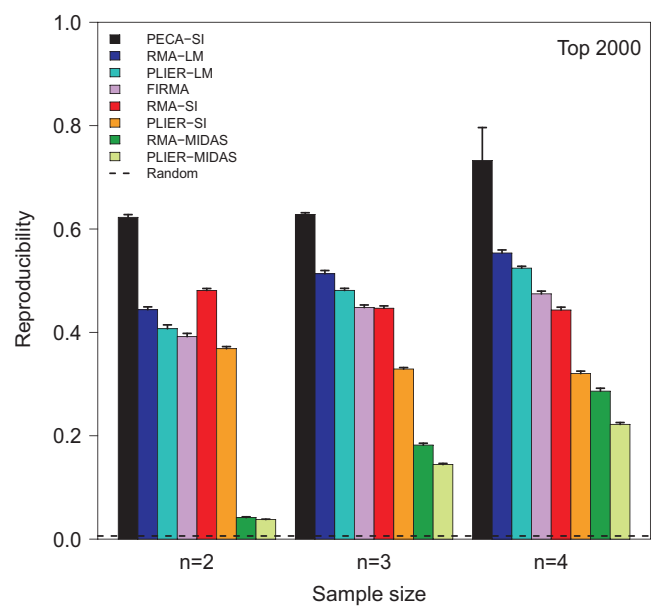

Supplement: Additional data file 2 — Reproducibility of PECA-SI, RMA-SI, PLIER-SI, RMA-MIDAS, PLIER-MIDAS, RM-LM, PLIER-LM and FIRMA in detecting differential splicing between laboratories. The ability of the methods to detect the same candidate splicing events in two independent hybridizations of the same biological samples was investigated at sample sizes two to four (x-axis). The reproducibility was measured as the overlap of the top-ranked k detections between the laboratories; k = 100, 1,000, 1,500, 2,000. At each sample size, the average reproducibility is shown together with the standard error of the mean (error bars). The same datasets were analyzed with the different detection methods. Reproducibility in random data is shown as a reference (0.0003, 0.003, 0.005 and 0.006 with the top 100, 1,000, 1,500 and 2,000 detections, respectively). [file gb-2009-10-7-r77-S2.pdf]

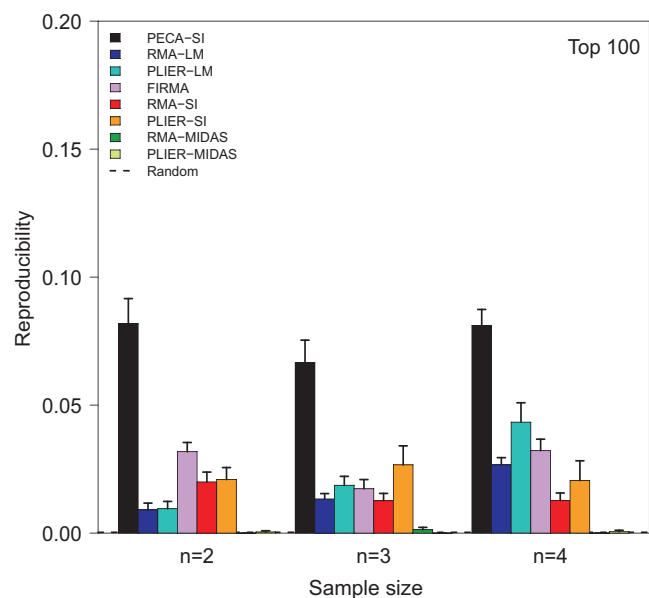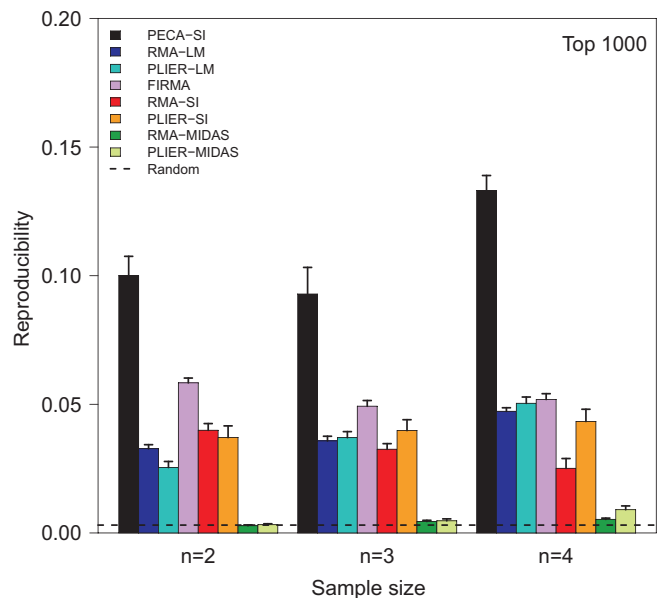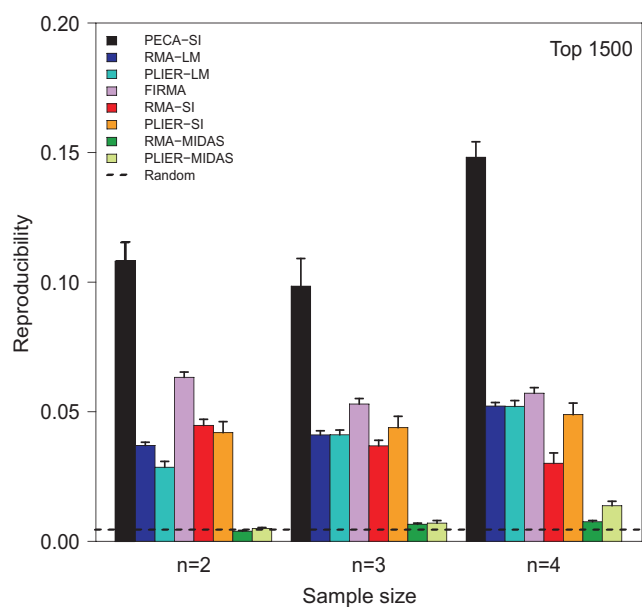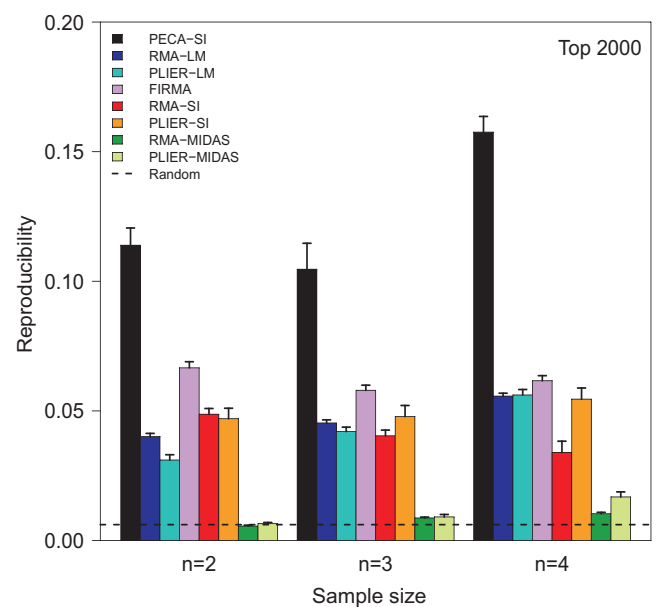

Supplement: Additional data file 3 — Reproducibility of PECA-SI, RMA-SI, PLIER-SI, RMA-MIDAS, PLIER-MIDAS, RM-LM, PLIER-LM and FIRMA in detecting differential splicing in the colon cancer data. The ability of the methods to detect the same candidate splicing events in independent subsamples was investigated at sample sizes two to four (x-axis). The reproducibility was measured as the overlap of the top-ranked k detections between the subsamples; k = 100, 1,000, 1,500, 2,000. At each sample size, the average reproducibility over at least 15 randomly sampled pairs of datasets is shown together with the standard error of the mean (error bars). The same datasets were analyzed with the different detection methods. Reproducibility in random data is shown as a reference (0.0003, 0.003, 0.005 and 0.006 with the top 100, 1,000, 1,500 and 2,000 detections, respectively). [file gb-2009-10-7-r77-S3.pdf]

(a)

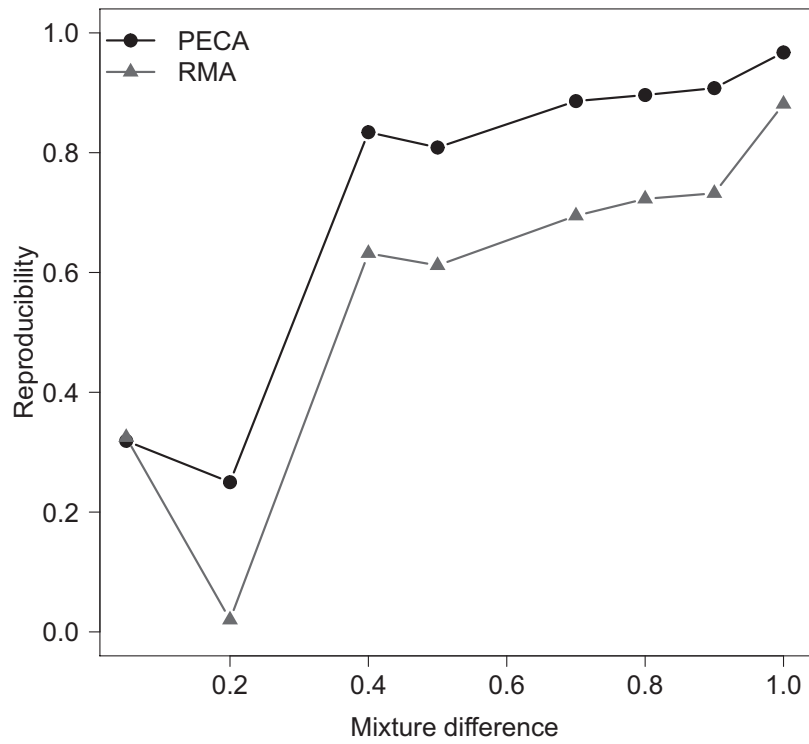

(b)

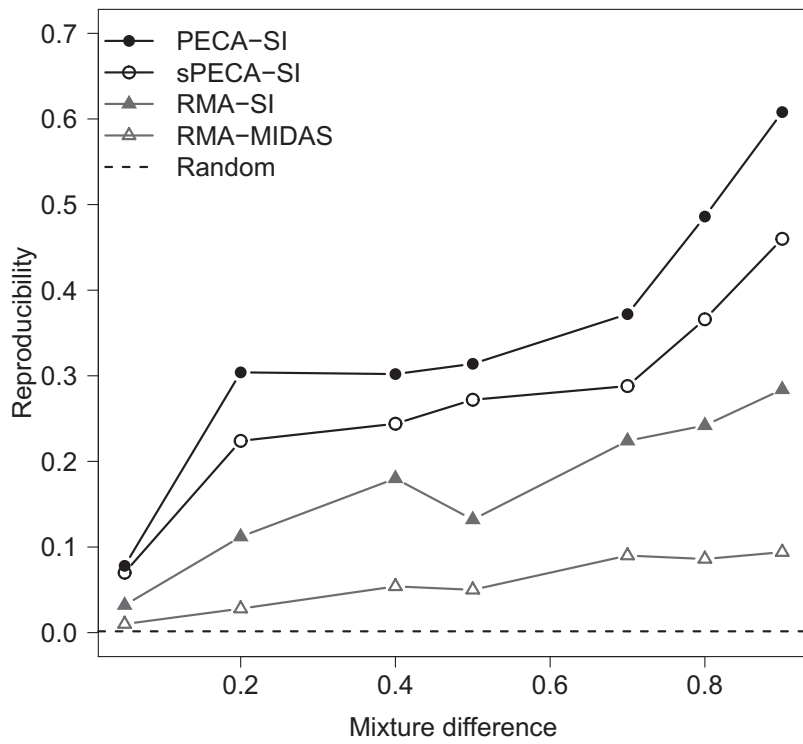

Supplement: Additional data file 4 — Illustration of the benefits gained by the probe-level estimation of PECA-SI in the mixture data. (a) Probe-level PECA improves the estimation of the gene-level signal log-ratios as compared to the standard procedure of using the RMA-normalized summary intensities. Each mixture contains three technical replicates, which are assumed to produce the same results across the arrays. To asses the performance of the PECA- and RMA-based estimation, we repeatedly calculated signal log-ratios between pairs of samples from distinct mixtures and then assessed the reproducibility of the estimates between the technical replicates using the Spearman correlation. The average reproducibility at various mixture differences (x-axis) is shown together with the standard error of the mean (error bars). It can be observed that the probe-level PECA produced systematically higher reproducibility values than RMA (paired Wilcoxon test, P < 10-6).(b) The probe-level PECA-SI improves the estimation of the exon-level statistic for differential splicing as compared to the standard summary intensity-based procedures (RMA-SI and RMA-MIDAS) or to a probe-level procedure, in which PECA is first applied separately to calculate the gene- and exon-level changes and these summarized changes are then used to calculate the statistic for SI (sPECA-SI). Similarly as in Figure 1, the ability of the methods to reproduce the detections from the pure brain and heart samples was studied at various levels of the mixture differences (x-axis). The reproducibility was measured as the overlap of the top-ranked 500 detections between the mixture and pure datasets. At each mixture difference, the same data were analyzed with the different detection methods. Reproducibility in random data is shown as a reference (0.002). [file gb-2009-10-7-r77-S4.pdf]
